# Preconditioning of bone marrow mesenchymal stem cells with hydrogen sulfide improves their therapeutic potential

**DOI:** 10.18632/oncotarget.11166

**Published:** 2016-08-09

**Authors:** Qun Zhang, Song Liu, Tong Li, Lin Yuan, Hansen Liu, Xueer Wang, Fuwu Wang, Shuanglian Wang, Aijun Hao, Dexiang Liu, Zhen Wang

**Affiliations:** ^1^ Department of Physiology, Shandong University School of Medicine, Jinan, Shandong 250012, P.R. China; ^2^ Department of Medical Psychology, Shandong University School of Medicine, Jinan, Shandong 250012, P.R. China; ^3^ Key Laboratory of the Ministry of Education for Experimental Teratology, Shandong Provincial Key Laboratory of Mental Disorders, Department of Histology and Embryology, Shandong University School of Medicine, Jinan, Shandong 250012, P.R. China

**Keywords:** hydrogen sulfide, bone marrow mesenchymal stem cells, transplants, brain-derived neurotrophic factor, vascular endothelial growth factor

## Abstract

Bone marrow mesenchymal stem cells (BMSCs) transplantation has shown great promises for treating various brain diseases. However, poor viability of transplanted BMSCs in injured brain has limited the therapeutic efficiency. Hypoxia-ischemic injury is one of major mechanisms underlying the survival of transplanted BMSCs. We investigated the mechanism of preconditioning of BMSCs with hydrogen sulfide (H2S), which has been proposed as a novel therapeutic strategy for hypoxia-ischemic injury. In this study, we demonstrated that preconditioning of NaHS, a H2S donor, effectively suppressed hypoxia-ischemic-induced apoptosis whereby the rise in Bax/Bcl-2 ratio. Further analyses revealed Akt and ERK1/2 pathways were involved in the protective effects of NaHS. In addition, NaHS preconditioning increased secretion of BDNF and VEGF in BMSCs. Consistent with *in vitro* data, transplantation of NaHS preconditioned BMSCs *in vivo* further enhanced the therapeutic effects of BMSCs on neuronal injury and neurological recovery, associated with increased vessel density and upregulation of BDNF and VEGF in the ischemic tissue. These findings suggest that H2S could enhance the therapeutic effects of BMSCs. The underlying mechanisms might be due to enhanced capacity of BMSCs and upregulation of protective cytokines in the hypoxia tissue.

## INTRODUCTION

A growing number of experimental studies highlights the potential of stem cell transplantation as a novel therapeutic approach for ischemic stroke [1]. Moreover, a variety of clinical trials have been performed and others are currently ongoing [2]. Bone marrow-derived mesenchymal stem cells (BMSCs) are widely used in cell therapy because of their ease of acquisition and expansion, immune tolerance, and differentiation capacity [3, 4]. Transplantation of BMSCs in the acute stage of ischemic stroke often modulates the inflammatory milieu, reduces lesion size and inhibits apoptosis in the penumbra area by providing neuroprotective paracrine factors [3, 5, 6]. However, only a small fraction of grafted cells survived in the ischemic brain 28 d after grafting because of the interplay of poor blood supply, ischemia reperfusion, inflammation, and apoptosis. Donor variability is another challenge for BMSC therapy trials.

Hydrogen sulfide (H_2_S) has been classified as a novel gasotransmitter signaling molecule in the central nervous system (CNS), involving in the regulation of ion channels, neurotransmitter functions, and other intracellular signaling molecules such as tyrosine kinases [7]. Interestingly, accumulating evidence has been garnered which suggests that exogenous H_2_S can function as a powerful neuroprotective agent. Kimura et al. demonstrated that H_2_S protected primary rat cortical neurons from oxidative stress-induced injury [8]. H_2_S exerts a number of cytoprotective and anti-inflammatory, anti-oxidant, and anti-apoptotic effects in CNS [9–11]. Recently, our findings showed the neuroprotective potential of H_2_S in animal models of cerebral hypoxia injury [12, 13]. Importantly, we observed that H_2_S was able to promote proliferation and neuronal differentiation of neural stem cells, and protect against hypoxia-induced decrease in hippocampal neurogenesis [14]. Although there is only limited information about anti-apoptotic effect of the endogenous H_2_S in BMSCs, the potential therapeutic value of H_2_S for BMSCs transplant has been increasingly recognized [15].

In this study, we tested the hypothesis that preconditioning with sodium hydrosulfide (NaHS), a H_2_S donor, enhances the survival of BMSCs upon exposure to hypoxia-ischemic insult *in vivo* and *in vitro*. We also sought to elucidate the underlying mechanisms of H_2_S preconditioning in BMSCs.

## RESULTS

### H_2_S preconditioning promotes proliferation of BMSCs under hypoxia-ischemic condition

**Figure 1:**
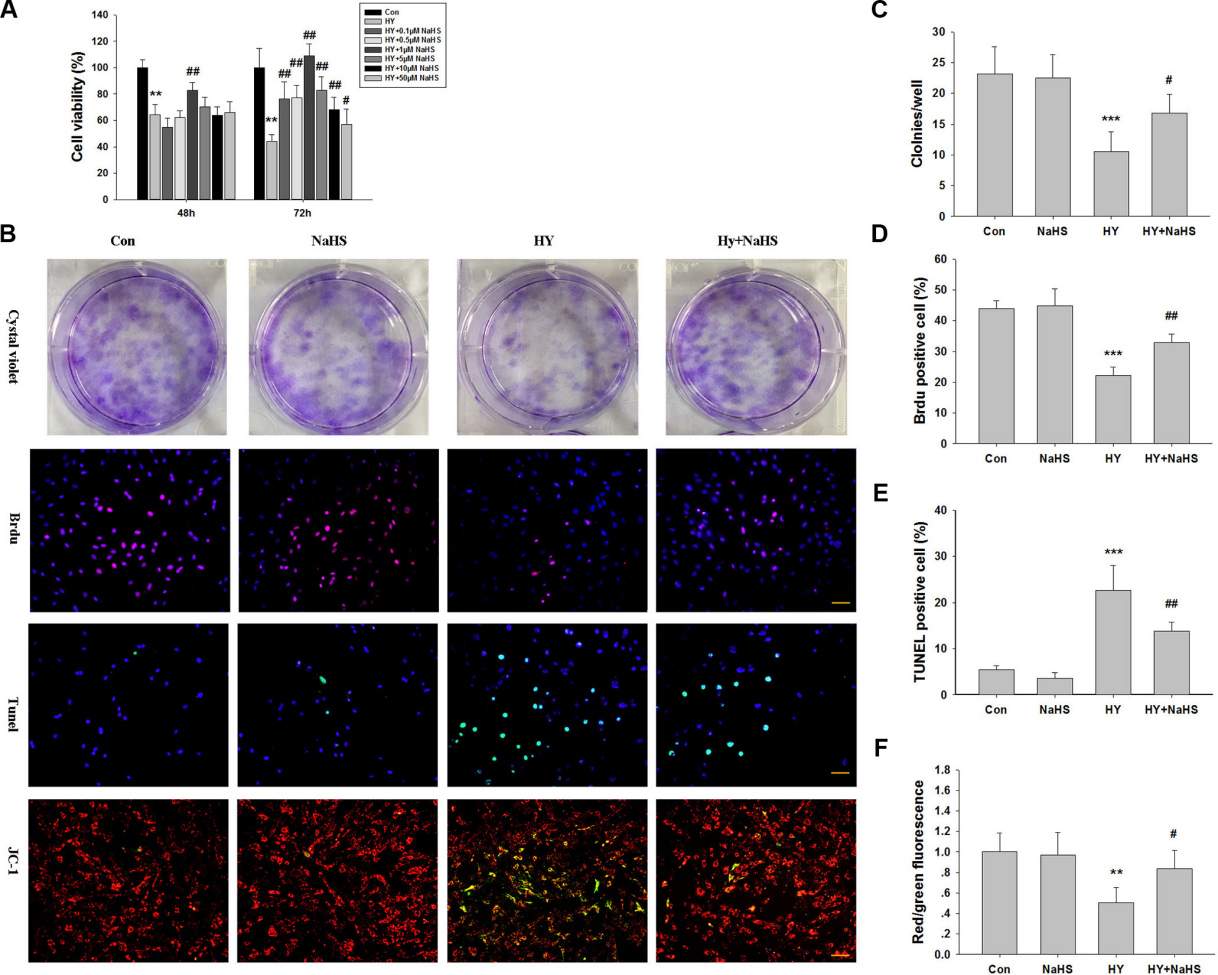
Effects of NaHS treatment on cell viability in bone marrow mesenchymal stem cells (BMSCs) *in vitro* (**A**) BMSCs maintained in 1% serum medium exposed to hypoxia (hypoxia-ischemic injury) were incubated in the absence or presence of indicated concentrations of NaHS (0.1–50 μM) for 48 h and 72 h and cell viability was examined by MTT assay. Values of cell viability were expressed as a percentage relative to those obtained in controls. Values represent the mean ± SD of *n* = 6. (**B**) BMSCs exposed to hypoxia-ischemic were incubated in the absence or presence of indicated concentrations of NaHS (1 μM) for 72 h. The cells were then subjected to crystal violet assay, BrdU assay (red), tunnel staining, and JC-1 staining, counterstained with DAPI (blue). Scale bar = 50 μm. (**C**) The graphs indicate the number of positive colonies/well by crystal violet staining, *n* = 6. (**D**) Quantification of BrdU positive BMSCs over the total DAPI-positive cells, *n* = 4. (**E**) Quantification of Tunnel positive BMSCs over the total DAPI-positive cells. Values represent the mean ± SD of *n* = 4. (**F**) Quantitative analysis of the ratio of red/green fluorescence, *n* = 6. Values represent the mean ± SD. **p < 0.01, ***p < 0.001 Hypoxia (Hy) VS Control (Con); #*p* < 0.05, ##*p* < 0.01 Hy+ NaHS VS Hy.

The effects of various concentrations of NaHS (0.1, 0.5, 1, 5, 10 and 50 μM) on viability of BMSCs were assessed. MTT results showed that NaHS preconditioned BMSCs (^PC^BMSCs) had a significant reduction in death, compared with the non-preconditioned BMSCs (^non-PC^BMSCs) after 48 h (*p* < 0.01) and 72 h (*p* < 0.01) hypoxia-ischemic injury. Furthermore, 1 μM NaHS preconditioning yielded the optimal effect on cell viability (82.8 ± 6.07 % vs 64.2 ± 7.77 % at 48 h, *p* < 0.01; and 108.9 ± 9.12 % vs 44.0 ± 5.24 % at 72 h, *p* < 0.01, respectively) (Figure 1A). Given the effectiveness of NaHS preconditioning at these concentrations (0.1, 1 and 5 μM), this protocol was used in the most of the subsequent *in vitro* experiments, unless otherwise stated. In addition, cell viability following treatment with NaHS alone at 0.1 μM (99.9 ± 8.64%), 1 μM (98.3 ± 9.79%) and 5 μM (104.6 ± 11.38%) was not significantly different from the control group (100 ± 9.75%) at 72 h. To further confirm this result, the number of colonies was counted by crystal violet staining (Figure 1B and 1C). The result indicated that the number of MSCs preconditioned with NaHS (1 μM) increased faster than that of hypoxia-ischemic exposure cells.

We then took BrdU staining assay to quantify the proliferating BMSCs, The result showed that the ratio of BrdU positive cells against total BMSCs were significantly decreased under the 72 h hypoxia-ischemic condition (*p* < 0.001), while the effects were significantly attenuated by co-treated with NaHS (1 μM) (Figure 1B and 1D, *p* < 0.01).

### H_2_S preconditioning suppresses apoptosis of BMSCs under hypoxia-ischemic condition

The TUNEL assay revealed that TUNEL-positive BMCSc were significantly increased under the 72 h hypoxia-ischemic condition (*p* < 0.001), while the effect of hypoxia-ischemic on apoptosis of cells was significantly attenuated by co-treated with NaHS (1 μM) (Figure 1B and 1E, *p* < 0.01).

Depolarization of the inner MMP is a sign of apoptosis [16]. Therefore, in order to ascertain whether NaHS preserves mitochondrial integrity through the maintenance of MMP, we performed JC-1 staining. As shown in Figure 3B, the red/green ratio of JC-1 was decreased in the BMSCs exposed to hypoxia-ischemic insult compared with the normal group, and this effect was reversed by NaHS (1 μM), which is consistent with the TUNEL assay (Figure 1B and 1F).

### H_2_S preconditioning decreased Bax/Bcl-2 ratio in BMSCs under hypoxia-ischemic condition

**Figure 2:**
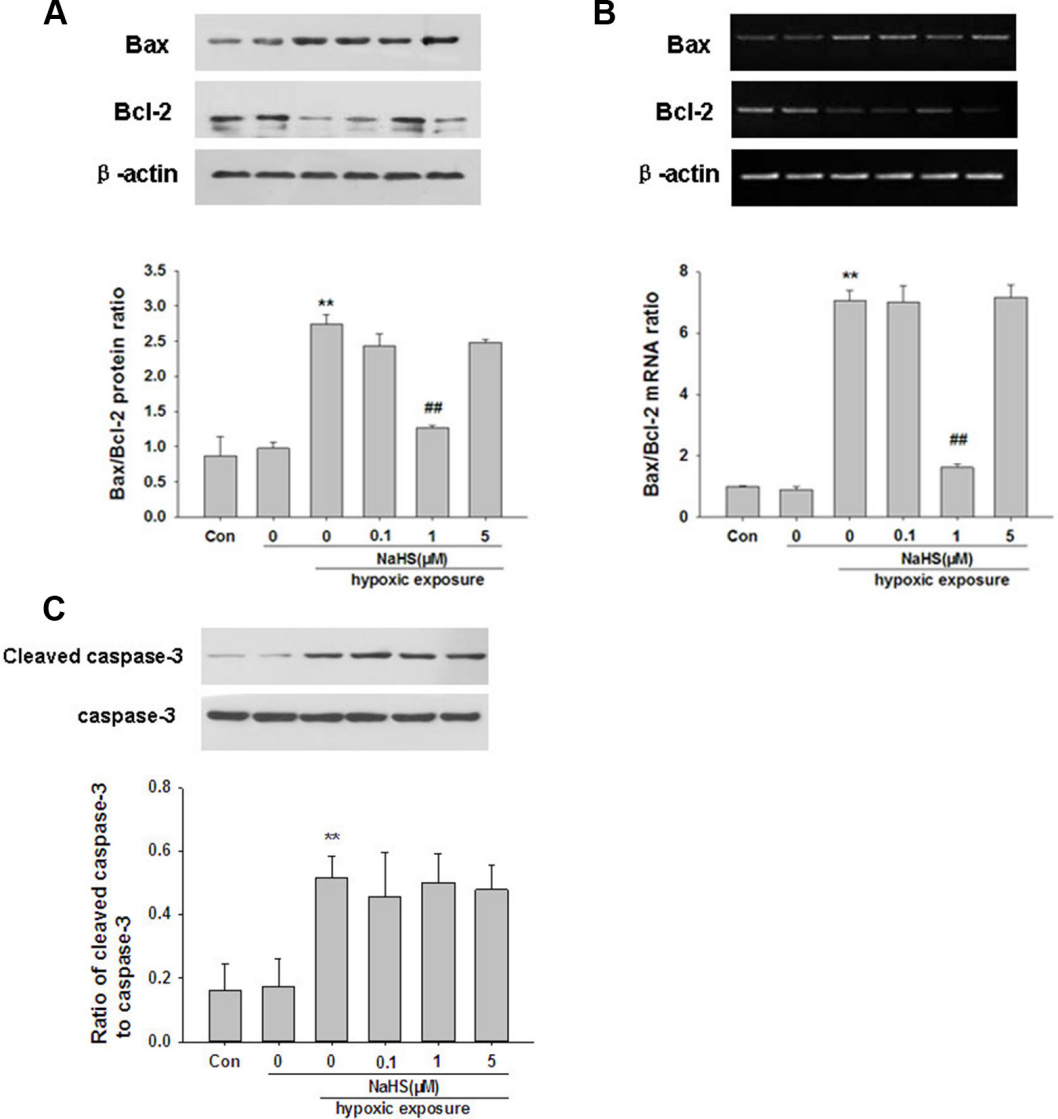
NaHS reverses hypoxia--ischemic induced changed of Bax and Bcl-2 and caspase-3 activation in BMSCs *in vitro* (**A**–**B**) BMSCs exposed to hypoxia-ischemic were incubated in the absence or presence of NaHS (1 μM) for 72 h. The protein and mRNA levels of Bax and Bcl-2 were then analyzed by Western blot (A) and by semiquantitative RT-PCR (B). Levels of β-actin were used to evaluate protein loading. Results were expressed as Bax/Bcl-2 ratio. (**C**) BMSCs exposed to hypoxia-ischemic were incubated in the absence or presence of NaHS (1 μM) for 72 h. The cells extracts were subjected to Western blot analysis using an antibody against cleaved caspase-3, Levels of caspase-3 were used to evaluate protein loading. Quantification of the protein levels of cleaved caspase-3 and caspase-3 as determined by Image-Pro Plus 6.0. Values represent the mean ± SD of *n* = 4. **p < 0.01 Hy VS Con; ##*p* < 0.01 Hy+ NaHS VS Hy.

Because changes in expression of pro-apoptotic Bax and anti-apoptotic Bcl-2 control the mitochondrial pathway of apoptosis, we examined the expression of Bax and Bcl-2 at the proteins and mRNA levels. As shown in Figure 2A and 2B, hypoxia-ischemic exposure markedly increased Bax, decreased Bcl-2, thereby increased the ratio of Bax/Bcl-2 at both protein and mRNA levels at 72 h post-injury (*p* < 0.001, *p* < 0.001, respectively). However, this signal increase was reduced by preconditioning with 1 μM NaHS (*p* < 0.001, *p* < 0.001, respectively). In addition, NaHS (1 μM) treatment alone did not significantly alter the Bax/Bcl-2 ratio.

### H_2_S preconditioning did not affect caspase-3 activation in BMSCs under hypoxia-ischemic condition

To further investigate the potential protective mechanism of NaHS, we performed Western blot to assess cleaved-caspase-3 protein levels after hypoxia-ischemic insult. As shown in Figure 2C, hypoxia-ischemic exposure significantly increased cleaved-caspase-3 protein levels (*p* < 0.01) compared with the corresponding controls. However, preconditioning with NaHS did not affect this hypoxia-ischemic induced cleaved-caspase-3 increase (*p* > 0.05).

### ERK1/2 and Akt pathway mediates the protection of H_2_S preconditioning in BMSCs under hypoxia-ischemic condition

**Figure 3:**
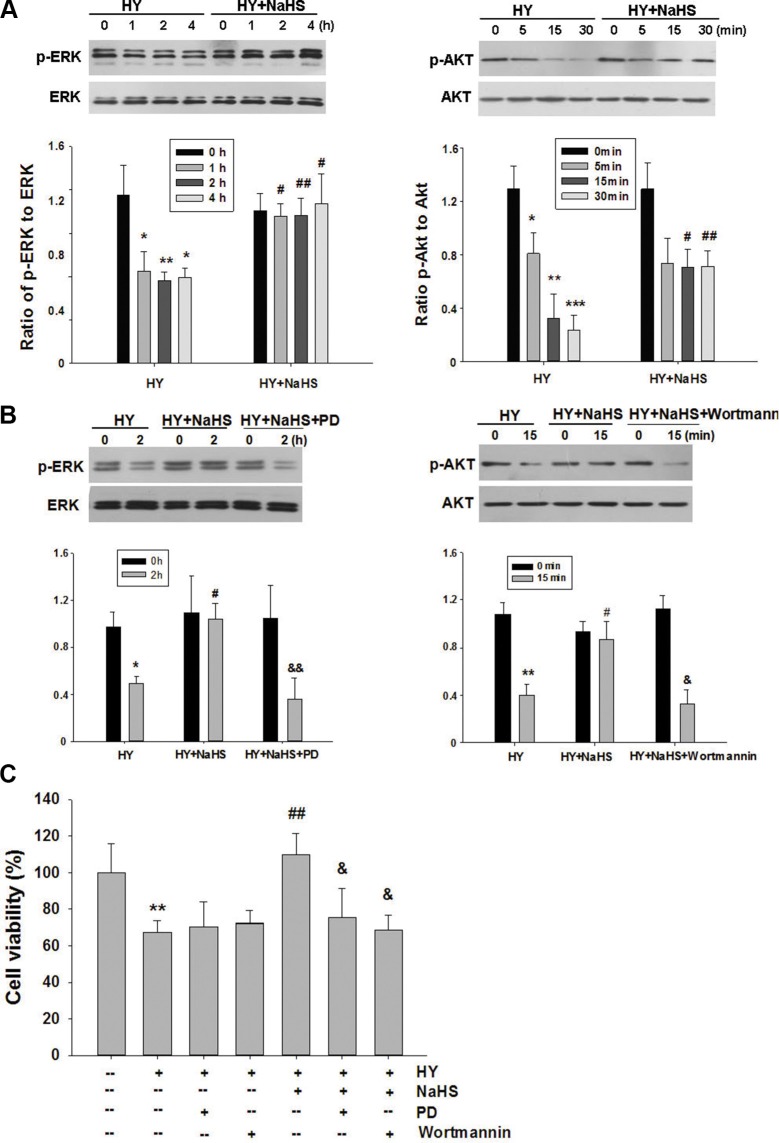
Effect of NaHS on ERK and Akt phosphorylation in BMSCs *in vitro* (**A**) BMSCs exposed to hypoxia-ischemic were incubated in the absence or presence of NaHS (1 μM) for indicated times and total protein was subjected to Western blot analysis. Bar graphs showing quantification of expression levels of phosphor-ERK/ERK or phosphor-Akt/Akt was determined by the Image-Pro Plus 6.0. *n* = 3. (**B**) BMSCs exposed to hypoxia-ischemic were incubated in the absence or presence of NaHS (1 μM), PD98059 (PD, 5 μM) or wortmannin (1 μM) for indicated times and total protein was subjected to Western blot analysis. Bar graphs showing quantification of expression levels of phosphor-ERK/ERK or phosphor-Akt/Akt was determined by the Image-Pro Plus 6.0. *n* = 3. (**C**) BMSCs were pretreated with PD98059 (PD, 5 μM) and wortmannin (1 μM) for 30 min, and then exposed to hypoxia-ischemic in the absence or presence of NaHS (1 μM) for 72 h. And cell viability was examined by MTT assay. Values of cell viability were expressed as a percentage relative to those obtained in controls, *n* = 6. Values represent the mean ± SD. *p < 0.05, **p < 0.01, ***p < 0.001 Hy VS Con; #*p* < 0.05, ##*p* < 0.01 Hy+ NaHS VS Hy; & *p* < 0.05, && *p* < 0.01 HY + PD/ HY + wortmannin VS HY.

The roles of ERK1/2 and Akt pathways upon the neuroprotective effects of H_2_S were assessed using Western blot analysis. As shown in Figure 3A, the levels of phosphorylation of ERK1/2 and Akt were decreased when exposed to hypoxia-ischemic insult. Preconditioning with 1 μM NaHS for 60, 120 and 240 min were all effective in up-regulating ERK1/2 activation in the hypoxia-ischemic group (Figure 3A). To implicate ERK1/2 in NaHS-induced protection against injury, BMSCs were treated with the specific blocker PD98059 to inhibit this pathway. PD98059 (5 μM) reversed the effect of NaHS on ERK1/2 activation in the hypoxia-ischemic group (Figure 3B). Moreover, analysis by MTT showed that PD98059 (5 μM) attenuated the anti-apoptotic effect of NaHS preconditioning after 72 h hypoxia-ischemic exposure (Figure 3C). In parallel with its effect on phosphorylation of ERK1/2, NaHS preconditioning for 5, 15 and 30 min caused a significant increase in Akt phosphorylation (Figure 3A). Administration of the potent PI3K inhibitor, wortmannin (1 μM), markedly reversed the effect of NaHS on Akt phosphorylation (Figure 3B), and blocked the anti-apoptotic actions of NaHS after 72 h hypoxia-ischemic exposure (Figure 3C).

### H_2_S preconditioning promotes BDNF and VEGF release from BMSCs

**Figure 4:**
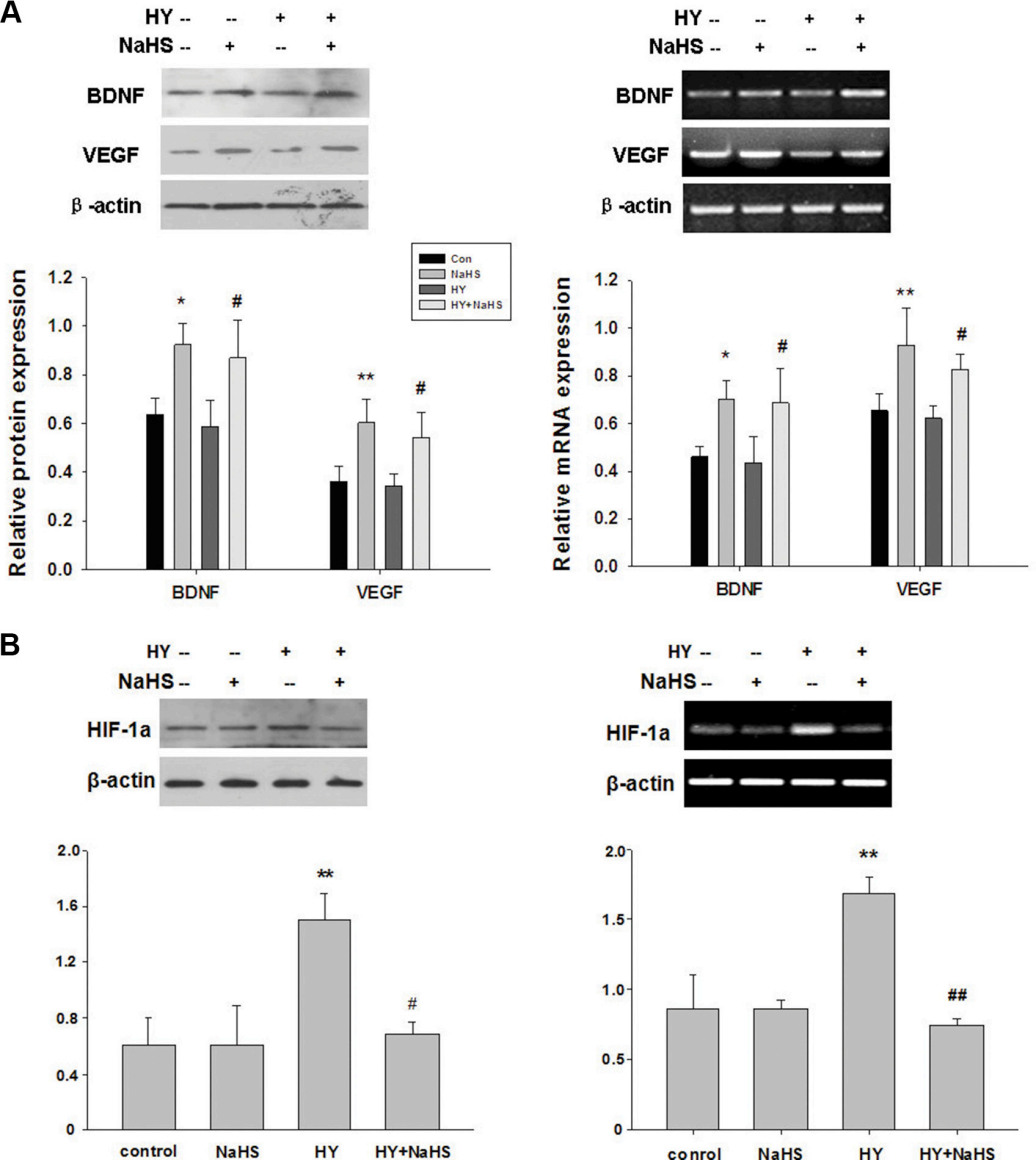
NaHS stimulated the release of BDNF and VEGF, and HIF-1a expression in BMSCs *in vitro* (**A**) BMSCs exposed to hypoxia-ischemic were incubated in the absence or presence of NaHS (1 μM) for 72 h. The mRNA and protein levels of BDNF, VEGF, and β-actin were then analyzed by western blot and semiquantitative RT-PCR. (**B**) BMSCs exposed to hypoxia-ischemic were incubated in the absence or presence of NaHS (1 μM) for 72 h. The mRNA and protein levels of HIF-1a and β-actin were then analyzed by western blot and semiquantitative RT-PCR, Each value was normalized to β-actin. Data was expressed as the mean ± SD of *n* = 4. *p < 0.05, **p < 0.01 NaHS VS Con; #*p* < 0.05, ##*p* < 0.01 HY + NaHS VS HY.

BMSCs have been shown to benefit neurological recovery after cerebral ischemia. The possible mechanisms are excreting trophic factors. Thus, we further investigated the effect of preconditioning BMSCs with NaHS (1 μM) on neuroprotective factors such as BDNF and VEGF. As shown in Figure 4A, NaHS treatment promoted RNA and proteins expression of BDNF and VEGF of BMSCs both in normal and hypoxia-ischemic conditions, compared with no NaHS treatment. Exposure to hypoxia-ischemic insult slightly decreased VEGF mRNA in comparison to control group while the differences were not statistically significant (Figure 4A, *p* > 0.05).

### H_2_S preconditioning inhibits hypoxia-induced HIF-1a expression in BMSCs

As shown in Figure 4B, hypoxia-ischemic exposure markedly increased HIF-1a at both protein and mRNA levels at 72 h post-injury (*p* < 0.01, *p* < 0.01, respectively). However, this signal increase was reduced by preconditioning with 1 μM NaHS (*p* < 0.05, *p* < 0.01, respectively). In addition, NaHS (1 μM) treatment alone did not significantly alter the HIF-1a level.

### Administration of ^PC^BMSCs improves a greater neurological function *in vivo*

**Figure 5:**
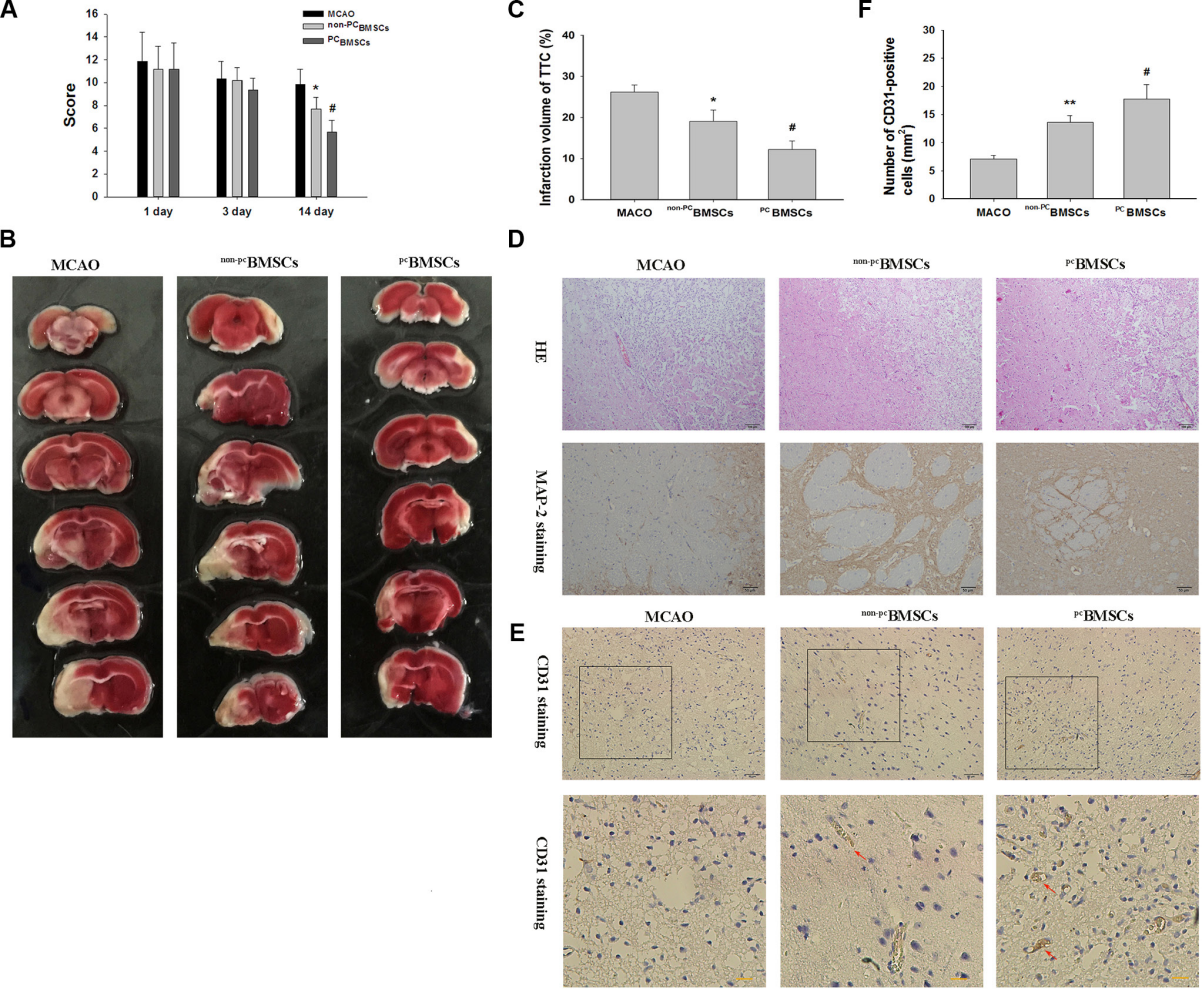
Administration of NaHS preconditioned-BMSCs (PCBMSCs) improved neurological function, decreased infarct volume, promoted neuronal density and MAP-2-positive cells in the infarct area (**A**) The scores of mNSS were measured at day 1, 3 and 14 after BMSCs transplantation in MACO group, ^non-PC^BMSCs group and ^PC^BMSCs group. *n* = 6. (**B**) Brain sections stained with TTC to visualize the ischemic lesions at day 14 after BMSCs transplantation in MACO group, ^non-PC^BMSCs group and ^PC^BMSCs group. (**C**) Quantitative analysis of the Infarct Volume. *n* = 3. (**D**) Representative photomicrographs (×10) of the infarct area by HE staining. Scale bar = 100 μm. *n* = 4. And Representative photomicrographs (×20) of MAP-2 immunohistochemistry in the infarct area. Scale bar = 50 μm. *n* = 4. (**E**) Representative photomicrographs of CD31 immunohistochemistry in the infarct area. Scale bar = 100 μm. (**F**) Quantitative analysis of CD31 positive cells. *n* = 4. Values represent the mean ± SD, *p < 0.05, **p < 0.05 ^non-PC^BMSCs VS MACO; #*p* < 0.05 ^PC^BMSCs VS ^non-PC^BMSCs.

To investigate the effects of ^PC^BMSCs in protecting neurons from hypoxia-ischemia injury *in vivo*, we established ischemic stroke models that were induced by MCAO in rats, and then transplanted ^PC^BMSCs into these rats intravenously. In the beginning, we evaluated neurological function using mNSS. The results showed there was no difference in mNSS among the no BMSCs group of rats (MACO group), ^non-PC^BMSCs group and ^PC^BMSCs groups at day 1 [F (2, 17) = 0.166, *p* > 0.05] and day 3 [F (2, 17) = 1.099, *p* > 0.05] after the transplantation. However, neurological deficits were improved in all groups at day 14 after transplantation [F (2, 17) = 20.043, *p* < 0.001]. Scores in ^non-PC^BMSCs group were lower than those in the sham control (*p* < 0.05). When compared with the ^non-PC^BMSCs group, transplantation with ^PC^BMSCs further reduced the scores (*p* < 0.05) (Figure 5A).

### Administration of ^PC^BMSCs reduces infarct volume following ischemic insults *in vivo*

The pale stained area was determined to indicate the infarct area (Figure 5B and 5C). The infarct volume in the MACO group (26.1% ± 1.72%) was significantly larger than that in the ^non-PC^BMSCs group (19.1% ± 2.76%, *p* < 0.05) at day 14 after transplantation. When compared with the ^non-PC^BMSCs group, transplantation with ^PC^BMSCs (12.2% ± 2.08%, *p* < 0.05) further reduced the infarct volume.

### ^PC^BMSCs transplantation reduces neuronal injury *in vivo*

Next we examined the cell morphology by HE staining. In the MACO group, cells were arranged sparsely and the cell outline was fuzzy in the infarct zone. Moreover, obvious edema was found in the infarct zone, and more necrotic cells were found in MACO group, while ^non-PC^BMSCs and ^PC^BMSCs transplantation alleviated edema and these morphological damages (Figure 5D). Importantly, the amelioration of cellular morphology was more significant in ^PC^BMSCs group than that in ^non-PC^BMSCs group. As shown in Figure 5D, the cell contour was distinguishable and nucleoli in the center of cells were observed clearly in ^PC^BMSCs group, and only a few neurons were shown to have nuclear pyknosis, hyperchromasia and extremely loose organization.

As shown in Figure 5D, MAP-2 immunolabeling was markedly diminished in the infarct zone, and there was an abrupt transition between the infarct and adjacent normal tissue with occasional MAP-2 positive cells in the transition zone in all groups. MAP-2 positive cells in the ^non-PC^BMSCs and ^PC^BMSCs transplantation group were more than those in the MCAO group. Moreover, MAP-2 positive cells in the ^PC^BMSCs group were significantly higher than those in the ^non-PC^BMSCs groups and (Figure 5D).

### Effect of ^PC^BMSCs on expression of CD31 in cerebral ischemia tissue after cerebral infarction

CD31 is mainly distributed in vascular endothelial cells and it is usually used to evaluate the vessel density in the tissue. As shown in Figure 5D and 5E, compared with the MACO group, the number of CD31-positive cells was increased in the ^non-PC^BMSCs group and ^PC^BMSCs (*p* < 0.01, *p* < 0.05, respectively) at day 14 after transplantation. The number of CD31-positive cells in the ^PC^BMSCs group was significantly higher than that of the ^non-PC^BMSCs (*p* < 0.05, respectively) (Figure 5E).

### ^PC^BMSCs induces anti-apoptotic effect on neuronal cells *in vivo*

**Figure 6:**
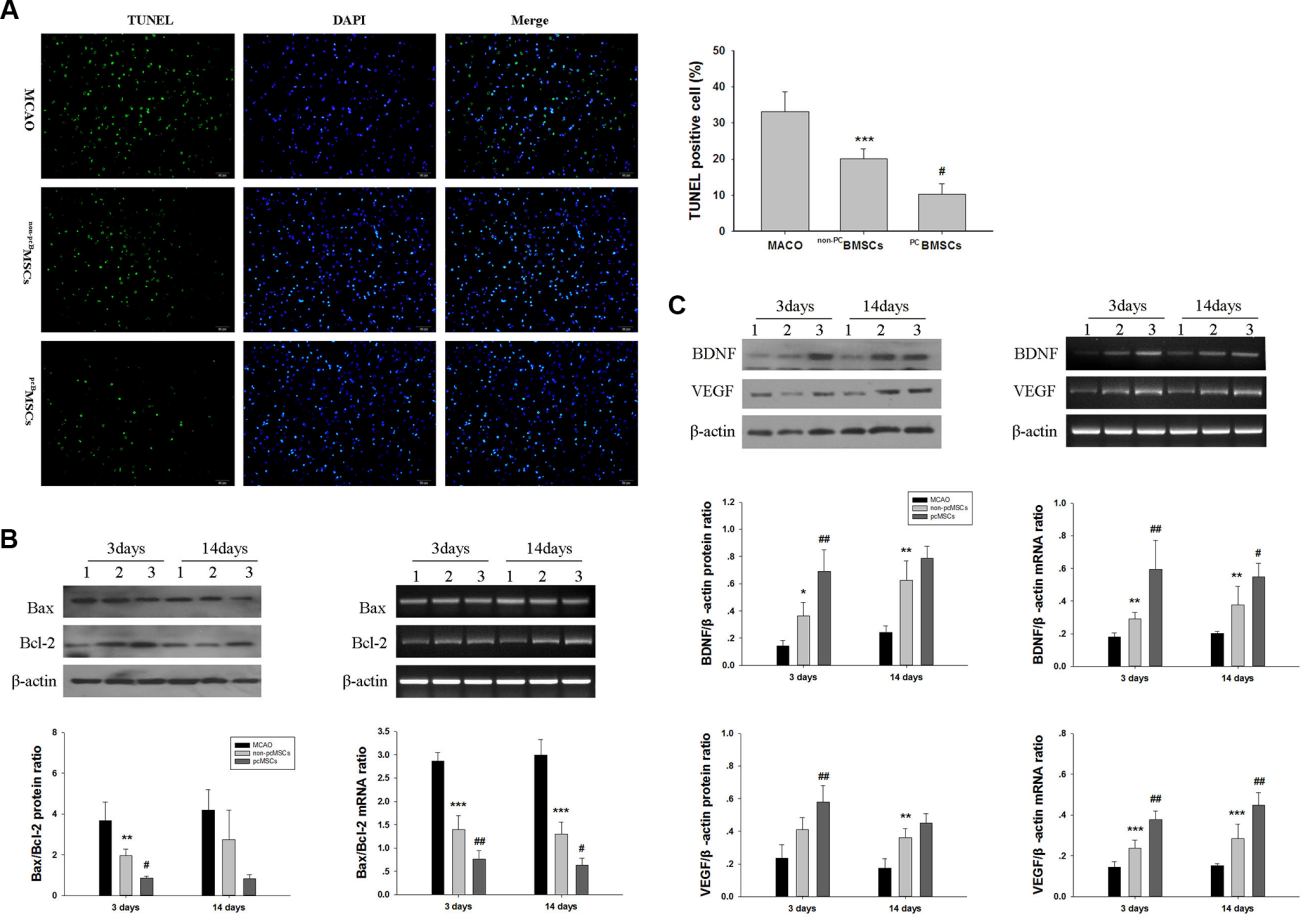
Administration of ^PC^BMSCs alleviated MACO-induced apoptosis, decreased Bax/Bcl-2 ratio and increased BDNF and VEGF expression (**A**) Representative photomicrographs (×20) of TUNEL staining of the brain tissues. Bar graphs showing quantification of TUNEL-positive cells. Scale bar = 50 μm. *n* = 4. (**B**) The protein and mRNA levels of Bax and Bcl-2 in injured cerebral tissues at day 3 and 14 after BMSCs transplantation were then analyzed by Western blot and semiquantitative RT-PCR, β-actin served as a loading control. Results were expressed as Bax/Bcl-2 ratio. *n* = 4. (**C**) The protein and mRNA levels of BDNF and VEGF in injured cerebral tissues at day 3 and 14 after BMSCs transplantation were then analyzed by Western blot and semiquantitative RT-PCR, β-actin served as a loading control. Each value was normalized to β-actin. *n* = 4.Values represent the mean ± SD *p < 0.05, **p < 0.01, ***p <0.001 ^non-PC^BMSCs VS MACO; #*p* < 0.05, ##*p* < 0.01 ^PC^BMSCs VS ^non-PC^BMSCs.

Ischemia induced significant TUNEL-positive cells in the ischemic penumbra at day 14 after MCAO (Figure 6A). Quantitative analysis demonstrated that the number of TUNEL-positive cells in the ^non-PC^BMSCs and ^PC^BMSCs group was lower than that in the MACO group (20.2% ± 2.67 vs 33.2% ± 5.39%, *p* < 0.001; 10.3 ± 2.90 vs 33.2% ± 5.39%, *p* < 0.001, respectively). Of note, the number of TUNEL-positive cells in the ^PC^BMSCs group was significantly lower than that of the ^non-PC^BMSCs groups (10.3 ± 2.90 vs 20.2 ± 2.67%, *p* < 0.05) (Figure 6A).

### ^PC^BMSCs decreases Bax/Bcl-2 ratio *in vivo*

The results demonstrated that compared with the MACO group, the ratio of Bax/Bcl-2 protein was decreased in the ^non-PC^BMSCs group (*p* < 0.01). The ratio of Bax/Bcl-2 protein in the ^PC^BMSCs group was significantly lower than that of ^non-PC^BMSCs groups (*p* < 0.05) (Figure 6B) at day 3 after transplantation. And there was no significantly difference between groups at day 14 after transplantation (*p* > 0.05) (Figure 6B). Similarly, the ratio of Bax/Bcl-2 mRNA was lower in the ^non-PC^BMSCs group when compared with that of the MACO group at day 3 and 14 after transplantation (*p* < 0.001, *p* < 0.001, respectively). The ratio of Bax/Bcl-2 mRNA in the ^PC^BMSCs group was significantly lower than that of the ^non-PC^BMSCs (*p* < 0.01, *p* < 0.05, respectively) (Figure 6B).

### ^PC^BMSCs improves BDNF and VEGF expression *in vivo*

The levels of BDNF protein in the ^non-PC^BMSCs group were significantly increased at day 3 and 14 after transplantation when compared with the MACO group (*p* < 0.05, *p* < 0.01, respectively) (Figure 6C). Moreover, transplanted with NaHS preconditioned BMCSs further upregulated expression of BDNF protein at day 3 after transplantation (*p* < 0.01) (Figure 6C). In addition, the levels of VEGF protein in the ^non-PC^BMSCs group were significantly increased at day 14 after transplantation when compared with the MACO group (*p* < 0.01). Transplanted with NaHS preconditioned BMCSs further upregulated expression of VEGF protein at day 3 after transplantation (*p* < 0.01), when compared with the ^non-PC^BMSCs group (Figure 6C).

As shown in (Figure 6C), ^non-PC^BMSCs treatment significantly increased mRNA levels of BDNF at day 3 and 14 after transplantation (*p* < 0.01, *p* < 0.01, respectively) and VEGF (*p* < 0.001, *p* < 0.001, respectively), as compared to the MACO group. ^PC^BMSCs groups treatment caused a further increase in the mRNA levels of BDNF at day 3 and 14 after transplantation (*p* < 0.01, *p* < 0.05, respectively) and VEGF (*p* < 0.01, *p* < 0.01, respectively), when compared to the ^non-PC^BMSCs group.

## DISCUSSION

We have shown in this study that H_2_S preconditioned BMCSs not only have a better survival rate than controls, but also express higher levels of BDNF and VEGF, resulting in stronger therapeutic effects in ischemic stroke. The major findings are: (a) H_2_S protected BMCSs from hypoxia-ischemic injury via down-regulation of Bax/Bcl-2 ratio *in vitro* and *in vivo*, (b) H_2_S preconditioning increased the BMCSs to release BDNF and VEGF *in vitro* and *in vivo*, (c) H_2_S increased the proliferation capacity of the BMCSs, via Akt and ERK1/2 pathways *in vitro*, (d) transplantation of the H_2_S preconditioned BMCSs attenuated infarct size and improved neurological recovery in MACO rats.

The mechanisms responsible for the cytoprotective benefits of H_2_S are not yet fully elucidated, but a number of suggestions have been provided. There are data which indicate that H_2_S exerts its cytoprotective benefits against excitotoxic insult as demonstrated both *in vitro* [17, 18] or *in vivo* in neurons [19, 20], and H_2_S also reduces cytotoxicity in astrocytes and microglia [21]. H_2_S exerts its anti-apoptotic effects through various mechanisms, including inhibition of cytochrome *c* release from mitochondria, normalized Bax /Bcl-2 levels, suppression of caspase-9/3 activation and poly(ADP-ribose) polymerase cleavage [7]. In this study, H_2_S preconditioning reduced apoptosis in the BMSCs and resulted in down-regulation of Bax/Bcl-2 ratio after hypoxia-ischemic insult.

Akt and ERK pathways are involved in numerous crucial cell functions, such as proliferation, differentiation, motility, survival, and intracellular trafficking [22, 23]. And previous studies have shown that H_2_S possesses neuroprotective effects in various models, some of which were dependent on the activity of the ERK and PI3K/Akt signaling pathway. For example, H_2_S therapy significantly suppressed caspase-3 activation and decreased myocardial injury via activating the ERK signaling pathway [24]. Furthermore, by activating the PI3K/Akt signaling pathway, H_2_S can increase Bcl-2 protein levels, inhibit activation of the cytochrome c-caspase-3/9 apoptosis pathway, and promote the survival of cells [25]. In line with these findings, our data indicate that H_2_S administration significantly enhanced Akt and ERK phosphorylation, and Akt or ERK inhibitor attenuated NaHS's promotion effects of cell survival, indicating that NaHS exerts its protective property in BMCSs after hypoxia-ischemic insult, at least in part, through the activation of Akt and ERK pathway.

Previous studies showed that intravenous administration of BMSCs after global cerebral ischemia decreased neural damage, suggesting that BMSCs increase neuron survival by intravenous infusion [6, 26, 27]. In this study, we found that BMSCs attenuated infarct size and improved the neurological deficits from day 14 after ischemic stroke and transplantation onward, indicating that neuroprotective effect is one of the main mechanisms of stem cell action in the present study. The paracrine factors produced by BMCSs such as VEGF [28], BDNF [29], and NGF [30] have a neuroprotective potential in animal ischemic stroke models. *In vitro* or *in vivo*, our results showed that BMSCs preconditioned by NaHS could enhance secretion of BDNF and VEGF, uniformly. In fact, previous studies have shown that H_2_S treatment induces VEGF and angiogenic factor after ischemic stroke and improves neurological outcomes [31]. In addition, H_2_S upregulates the level of BDNF protein and blockage of BDNF-TrkB pathway reverses the protection of H_2_S against cytotoxicity, oxidative stress, and apoptosis in neurons [32, 33]. Importantly, to determine whether the bioactive factors released from H_2_S-preconditioned BMSCs provided protective effects on neurons subjected to injury, we treated MCAO animals with NaHS (1 μM) via tail vein in preliminary studies, the results showed that NaHS administration alone did not protect neurons from hypoxia-ischemia injury *in vivo*. Taking together these data and the results of the present study, we hypothesize that enhancement of paracrine effects of BMSCs for these neuroprotective factors may be indirectly resulted from improvement of the transplanted BMSCs survival due to preconditioning with H_2_S.

Angiogenesis can improve the micro-circulation in the zone of cerebral ischemia and inhibit the cell apoptosis. Moreover, the new blood vessels can induce the release of nutritional factors to promote the recovery of neurological function. In this study, we found that H_2_S-preconditioned BMSCs can promote the angiogenesis after cerebral infarction by CD31 staining, associated with enhanced secretion of VEGF. These results are similar to those in the previous studies [34, 35]. Thus it can be presumed that the reason for H_2_S-preconditioned BMSCs to recover the neurological function of animals with cerebral infarction may be the action to promote the angiogenesis. In addition, we found that hypoxia do not increase the VEGF expression in BMSCs, which is in contradiction with a numerous number of previous data [36, 37]. The discrepancy might be due to the severity of the insult and the time used in these studies.

In the cerebral ischemia, HIF-1α was increased and it induced the expression level of its downstream target genes, such as VEGF, erythropoietin, and CXC chemokine receptor 4 [38]. HIF-1α is also involved in angiogenesis, cell survival, anaerobic metabolism, cell migration, and differentiation, suggesting that HIF-1α plays an important role in the functional recovery of cerebral ischemia [38]. Previous studies showed that H_2_S increased HIF-1α and VEGF expression in vascular smooth muscle cells under hypoxic condition, which enhanced endothelial cell viability [39, 40]. Recently, one study showed that HIF-1α-induced VEGF overexpression in BMSCs protected neuron against brain ischemia [36]. Our findings are in agreement with these previous reports and revealed that BMSCs preconditioned by NaHS could enhance secretion of HIF-1α and VEGF, associated with a decreased apoptotic rate, suggesting that the NaHS-preconditioned BMSCs promote cellular survival through the regulation of VEGF by HIF-1α.

In this regard, it is important to note that a recent study indicated that therapeutic efficacy of rat BMSCs preconditioned with 200 μM NaHS added after myocardial ischemia in Sprague-Dawley rats [41], thus the used cells received much higher dose of H_2_S compared to the cells applied in our study. In our study NaHS at lower concentration can improve the survival of stem cells used for cell-therapy in the context of an *in vitro* cell therapy. Similar results were reported by other authors, who demonstrated that NaHS at 3 μM pretreatment can increase the survival of therapeutically used human adipose tissue-derived stem cells [42]. There are several important differences between these investigations. For example, in this study BMSCs were challenged to hypoxia for 72 h in 1% FBS medium, for the BMSCs failed to proliferate in serum-free conditions [43].

There are some important limitations to our study. First, whether H_2_S preconditioning change property of BMCSs which differentiate into neuronal lineage cells was not investigated. Second, how H_2_S up-regulates expression of BDNF and VEGF, and how these cytokines affect the neurological function were not determined. Finally, how the effects of H_2_S preconditioning using other routes/timing of stem cell delivery were not evaluated.

In conclusion, we have demonstrated that H_2_S preconditioning presented better neuron protection and an improvement of neurological function under hypoxic- ischemic conditions, which might provide a better therapy of BMSCs administration to cerebral ischemia. We have also elucidated the possible mechanisms of the beneficial effects of H_2_S preconditioned BMSCs acting, at least in part, through up-regulation of the survival of BMSCs and secretion of paracrine factors.

## MATERIALS AND METHODS

### Bone marrow mesenchymal stem cell cultures

Bone marrow mesenchymal stem cells (BMSCs) were harvested from 3 weeks Wistar rats as previously described [44]. Briefly, BMSCs were flushed out from the femoral and tibial bones of donor adult rats using a syringe and 20-gauge needle. The cells were suspended in DMEM/F12 supplemented with 10% FBS and incubated in 95% room air and 5% carbon dioxide at 37°C and nonadherent cells were removed by replacing the medium. The primary cultures were passaged at a ratio of 1:3 once the BMSCs reached 80–90% confluence. At passage 3, BMSCs were used for the present study.

### Hypoxia-ischemic protocol

Prior to use in the experiment, plated cells were incubated with low serum (1% FBS) for 1 h. The medium was then replaced with low serum DMEM containing NaHS (Sigma-Aldrich) for the various time intervals and concentrations as indicated below. PI3K inhibitors (Wortmannin) and ERK1/2-MAPK inhibitor (PD98059) were purchased from Sigma-Aldrich.

BMSCs were challenged to hypoxia by placing them in a chamber (Model: Heraeus HERAcell 240i; Thermo Scientific, USA) filled with 3% oxygen, 5% CO_2_ and 92% nitrogen at 37°C for the time intervals indicated below. BMSCs serving as controls were incubated at 37°C with 95% air and 5% CO_2_. Three or more different donor BMSCs were evaluated in triplicate for all experiments.

### Cell viability assay

Cell viability was determined by the MTT assay. BMSCs were plated into 96-well culture plates at a density of 5 × 10^4^ cells/ml with 200 μl culture medium per well. Following exposure to hypoxia with or without differing concentrations of NaHS, 20 μl MTT solution (5 mg/ml) was added to each well and incubated for 4 h. The medium was then aspirated and 200 μl dimethyl sulfoxide was added. The absorbance value was measured using a multiwell spectrophotometer (Bio-Rad, USA) at 490 nm. Cell viability was expressed as a percentage of viable cells obtained relative to that of controls.

### Crystal violet assay

BMSCs were treated with test substances or vehicle for 72 h, and then fixed with 4% paraformaldehyde, stained with 0.5% crystal violet in 4% paraformaldehyde. The number of colonies was then counted. Colonies of less than 2 mm in diameter and faintly stained colonies were ignored [45].

### Proliferation index by bromodeoxyuridine (BrdU) labeling

Cell proliferation was evaluated by BrdU incorporation assay. Dissociated BMSCs were transferred into 12-well plates with poly-D-lysine coated coverslips in medium. After this, BMSCs were treated with test substances or vehicle for 72 h and incubated with BrdU (10 μmol/l, Sigma-Aldrich) for 2 h. The cultured cells were fixed in 4% paraformaldehyde for 20 min, treated with 2 mol/l HCl at 37°C for 30 min, blocked with 10% normal goat serum for 30 min, and incubated with anti-BrdU monoclonal antibody (1:250; Sigma-Aldrich) overnight at 4°C. Goat anti-mouse IgG coupled to tetramethyl rhodamine iso-thiocyanate (TRITC) (1:200; Chemicon, Temecula, CA, USA) was used as the secondary antibody. The percentage of BrdU-positive cells over total DAPI cells was determined by randomly counting 10-nonoverlapping microscopy fields from each condition, in at least four independent experiments. Counting was performed in a blinded manner.

### Apoptosis analysis *in vitro*

The TUNEL assay for the detection of morphological features of apoptotic cell death was performed by using the *in situ* cell detection kit (FITC) following the manufacturer's instructions (Chemicon, Temecula, CA, USA). The features of apoptosis were examined, as described previously [46]. Images of TUNEL-positive cells (with a green fluorescent nucleus) were captured with a fluorescence microscope (IX71; Olympus, Tokyo, Japan) and randomly counted in 10-nonoverlapping microscopy fields from each experimental condition, in at least four independent experiments. The proportion of TUNEL-positive cells was expressed as the percentage of the total cells counted. Counting was performed in a blinded manner.

### Detection of mitochondrial membrane potential (MMP or ΔΨm)

The loss of ΔΨm was determined using the JC-1 Mitochondrial Membrane Potential assay kit following the manufacturer's instructions (Beyotime Institute of Biotechnology, China). Briefly, BMSCs were treated with test substances or vehicle for 72 h. Following treatment, BMSCs were washed with PBS and added JC-1 (5 μM), followed by incubation at 37°C for 20 min. Subsequently, BMSCs were washed twice with cold JC-1 staining buffer and visualized under a fluorescence microscope. The ratio of red to green fluorescence intensity was measured and data were expressed as relative to the mean sham value.

### Ischemia model of middle cerebral artery occlusion

Adult male Wistar rats (270 to 300 g) were used for ischemic stroke experiments. A middle cerebral artery occlusion (MCAO) was induced by a modification of the intraluminal vascular occlusion method as previously described [47]. Briefly, Rats were initially anesthetized with 10% chloral hydrate. Rectal temperature was controlled at 37°C with a feedback-regulated water heating system. The right common carotid artery, external carotid artery (ECA), and internal carotid artery were exposed. A 3.0 monofilament nylon suture (18.5 mm, determined by animal weight), with its tip rounded by heating near a flame, was advanced from the ECA into the lumen of the internal carotid artery until it blocked the origin of the middle cerebral artery. At 2 h after occlusion, the animals were re-anesthetized and reperfused by withdrawing the suture until its tip cleared the lumen of the ECA.

### Intravenous administration of BMSCs

NaHS was added to the cell culture medium (final concentration: 1 μM) for 72 h, followed by drug washout before transplantations. Then the deposits of single cell suspension in PBS (2 × 10^6^ cells per ml) was pre-incubated with or without NaHS (final concentration: 1 μM) for 30 min before transplantation.

After 2 h MCAO and 24 h reperfusion, rats were randomly divided into three groups: MACO group (rats given MCAO without cell administration but with 1 ml PBS), ^non-PC^BMSCs group (rats transplanted with 2 × 10^6^ BMSCs), and ^PC^BMSCs group (rats transplanted with 2 × 10^6^ NaHS preconditioning-BMSCs) via tail vein. For intravenous injection, BMSCs (2 × 10^6^) diluted in 1 ml PBS were injected slowly for five minutes in the tail vein. All transplantation procedures were performed under aseptic conditions.

### Behavioral tests

All animals were assessed with modified neurological severity score (mNSS) tests [48] (Table 1) at day 1, 3 and 14 after BMSCs transplantation (*n* = 6 for each group). Neurological function including motor and sensory systems as well as reflexes and a balance test is graded on a numeric scale from 0 to 18 (normal score, 0; maximal deficit score, 18); the higher the score, the more severe the neurological deficit.

**Table 1:** Modified neurological severity score (mNSS)

	Points
Motor tests	
Raising rat by the tail	3
1 Flexion of forelimb	
1 Flexion of hindlimb	
1 Head moved > 10° to vertical axis within 30 s	
Placing rat on the floor (normal = 0; maximum = 3)	3
0 Normal walk	
1 Inability to walk straight	
2 Circling toward the paretic side	
3 Fall down to the paretic side	
Sensory tests	2
1 Placing test (visual and tactile test)	
2 Proprioceptive test (deep sensation, pushing the paw against the table edge to stimulate limb muscles)	
Beam balance tests (normal = 0; maximum = 6)	6
0 Balances with steady posture	
1 Grasps side of beam	
2 Hugs the beam and one limb falls down from the beam	
3 Hugs the beam and two limbs fall down from the beam, or spins on beam (> 60 s)	
4 Attempts to balance on the beam but falls off (> 40 s)	
5 Attempts to balance on the beam but falls off (> 20 s)	
6 Falls off: No attempt to balance or hang on to the beam (< 20 s)	
Reflexes absent and abnormal movements	4
1 Pinna reflex (head shake when touching the auditory meatus)	
1 Corneal reflex (eye blink when lightly touching the cornea with cotton)	
1 Startle reflex (motor response to a brief noise from snapping a clipboard paper)	
1 Seizures, myoclonus, myodystony	
Maximum points	18

One point is awarded for the inability to perform the tasks or for the lack of a tested reflex; 13 to 18 indicates severe injury; 7 to 12, moderate injury; 1 to 6, mild injury.

### Cerebral infarction measurement

Cerebral infarction volume was measured by staining brain slices with triphenyltetrazolium chloride (TTC; Sigma) at day 14 after BMSCs transplantation. The animals (*n* = 3 for each group) were decapitated under deep anesthesia with 10% chloral hydrate (0.8 g/kg, i.p.). The brains were quickly removed and sliced into 6 coronal sections. The slices were incubated in a 2% solution of TTC at 37°C for 30 min and then fixed in a 10% formaldehyde solution overnight. The infarct area of each brain slice was analyzed using the Image-Pro plus 6.0 analysis software. The total infarction volume for each brain was calculated by summation of the infarcted area of all brain slices.

### Hematoxylin and eosin staining

At day 14 after BMSCs transplantation, animals were perfused under deep anesthesia with 10% chloral hydrate followed by 4% paraformaldehyde. The brains were then removed and post-fixed in formalin. After fixation and dehydration with gradient ethanol, the brain tissues were embedded in paraffin and sliced into in the coronal plane at 4 μm thickness using a section cutter (Leica, Germany). The sections (3 sections/rat) were stained with hematoxylin and eosin (H&E). And 4 rats for each group were prepared for HE staining. Morphology of ipsilateral cortex was observed by a light microscope (Olympus Corporation, Japan).

### Immunohistochemistry

The sections were dewaxed with a standard procedure and washed with PBS as described previously. Briefly, after blocking in 10% normal goat serum in PBS for 30 min at room temperature, the sections were incubated with anti-microtubule- associated protein 2 (MAP-2) (1:200; mouse monoclonal, Millipore Corporation, Billerica, MA, USA), CD 31(1:200; rabbit polyclonal, Cell Signaling Tech. MA, USA) at 4°C overnight. After primary antibody incubation, samples were washed and incubated with a goat anti-mouse/rabbit secondary antibody for 2 h at room temperature. The sections were washed and then incubated with an avidin biotinylated enzyme complex for 1 h at room temperature. The sections were visualized with diaminobenzidine. Nuclei were counterstained with hematoxylin. Finally, the sections were dehydrated in an alcohol gradient and cleared with xylene. The number of CD31-positive cells was counted in penumbra of hemisphere with the lesion under the light microscope (20×). For each section, five visual fields in penumbra of hemisphere with the lesion were chosen at random for statistical analysis (*n* = 4 each group). Results were expressed as the mean number of CD31 positive cells per mm^2^.

### TUNEL staining *in vivo*

The sections were dewaxed with a standard procedure and washed with PBS as described previously. TUNEL staining was done with commercially available kit as above described. The slides were counterstained with DAPI for total nuclei counting. The number of total and TUNEL-positive cells in the ischemic penumbra was calculated by counting six randomly selected microscopic fields in sections obtained from each rat (n = 4) at ×20 objective by a blinded observer. The percentage of TUNEL-positive cells against the total number of cells was calculated and averaged.

### Reverse transcription–polymerase chain reaction (RT-PCR)

Total RNA was extracted from cells using the Trizol reagent (Gibco, Invitrogen) according to the manufacturer's instructions. RNA concentration was determined using a spectrophotometer (Bio-Rad. Labs) at 260 nm. Identical amounts of RNA (2 μg) were reversely transcribed into cDNA using a commercial RT-PCR kit (Fermentas, Vilnius, Lithuania) according to the manufacturer's instructions. cDNA was subsequently amplified by PCR with specific primers (Table 2). PCR products, separated on a 1.2% agarose/TAE gel, were visualized by staining with ethidium bromide. The densitometric calculations of these values were normalized to β-actin. The intensity of bands was determined using Image-Pro Plus 6.0 software.

**Table 2:** PCR primers used in this study

*Gene*	*Forword (5′→3′)*	*Reverse (5′→3′)*
Bax	GGT TGC CCT CTT CTA CTT TGC	TCT TCC AGA TGG TGA GCG AG
Bcl-2	GGA TGA CTT CTC TCG TCG CTA C	TGA CAT CTC CCT GTT GAC GCT
BDNF	AGC TGA GCG TGT GTG ACA GT	ACC CAT GGG ATT ACA CTT GG
VEGF	GTG GAC ATC TTC CAG GAG TA	TCT GCA TTC ACA TCT GCT GT
β -actin	CTA TTG GCA ACG AGC GGT TCC	CAG CAC TGT GTT GGC ATA GAG G

### Western blot analysis

Protein concentration in the supernatants of cells or ipsilateral cortex was determined using a BCA protein assay kit (Pierce Biotechnology, Inc.). A quantity of 30–50 μg of total proteins was loaded onto a 10–15% gradient polyacrylamide gel, electrophoretically transferred to a polyvinylidene difluoride membrane and probed with the following primary antibodies: Bax antibody (1:1000, Santa Cruz Biotechnology, CA, USA), Bcl-2 antibody (1:1000, Santa Cruz Biotechnology), Cleaved caspase-3 (1:500, Cell Signaling Tech. MA, USA), Caspase-3 (1:1000, Cell Signaling), Phospho-extracellular signal-regulated kinase (ERK)1/2 (1:2000, Cell Signaling), ERK1/2 (1:2000; Cell Signaling), Phospho-Akt (Ser 473) (1:500, Cell Signaling), Akt (1:1000; Cell Signaling), Brain-derived neurotrophic factor (BDNF, 1:1000, Santa Cruz Biotechnology), Vascular endothelial growth factor (VEGF, 1:100, Santa Cruz Biotechnology). β-actin (1:2000; Sigma-Aldrich) was used as an internal control. Secondary antibodies were horseradish peroxidase conjugated to goat/mouse anti-rabbit IgG (1:8000, Sigma-Aldrich). The membranes were developed using an enhanced chemiluminescence detection system (Pierce, Rockford, IL).

### Statistical analysis

Quantitative data were presented as the mean ± SD. Statistical analysis of data was performed with a one-way ANOVA using the *post-hoc* Tukey test for multiple comparisons of means. Differences were considered statistically significant if the *p* value was < 0.05.

## Abbreviations

BDNF: brain-derived neurotrophic factor
BMSCs: Bone marrow mesenchymal stem cells
CNS: Central nervous system
DAPI: 4′ 6-Diamidino-2-phenylindole dihydrochloride
DMEM: Dulbecco's modified Eagle medium
ELISA: Enzyme-Linked Immunosorbent Assay
ERK: extracellular signal-regulated kinase
FBS: fetal bovine serum
H2S: hydrogen sulfide
MTT: 3-[4 5-dimethylthiazol-2-yl] −2 5-diphenyltetrazolium bromide sodium
MAPK: mitogen-activated protein kinase
NaHS: sodium hydrosulfide
PBS: phosphate buffered saline
RT-PCR: reverse transcription–polymerase chain reaction
VEGF: vascular endothelial growth factor
